# Measuring workplace gender microaggressions in Spain: validation and measurement invariance of the Spanish version of the MIMI-16

**DOI:** 10.3389/fpsyg.2026.1821085

**Published:** 2026-05-08

**Authors:** Gonzalo Guillem, Eva Cifre, Timo Lorenz, Mona Algner

**Affiliations:** 1 Departamento de Psicología Evolutiva, Educativa, Social y Metodología, Universitat Jaume I, Castellón de la Plana, Spain; 2 Department of Psychology, Medical School Berlin, Berlin, Germany

**Keywords:** confirmatory factor analysis, gender microaggressions, scale validation, sexism, women at work

## Abstract

**Introduction:**

Workplace gender microaggressions constitute subtle forms of symbolic violence that are often difficult to recognize, particularly in their manifestations as microinsults and microinvalidations. Although related constructs such as micromachismos and sexism have been examined in Spanish-speaking contexts, no validated Spanish-language instrument has operationalized these experiences in line with the microinsults–microinvalidations taxonomy. This study examines the psychometric properties of the Spanish adaptation of the Microinvalidations and Microinsults Scale (MIMI-16) in working women.

**Methods:**

Two independent samples of working women in Spain (*N*_1_ = 352; *N*_2_ = 404) were analyzed. Confirmatory factor analyses using a robust weighted least squares estimator (WLSMV), multigroup analyses for measurement invariance, and correlational analyses for criterion-related validity were conducted.

**Results:**

Supported the expected correlated two-factor structure, with moderate to high standardized loadings (*λ* = 0.60–0.92) and high reliability (*ω* = 0.88–0.89; composite reliability = 0.93–0.95). Measurement invariance was supported at the configural, metric, and scalar levels. Criterion-related validity was evidenced by associations with poorer workplace social relations, higher psychological distress and burnout, lower work engagement and job satisfaction, and greater turnover intention. However, the high inter-factor correlation and partial limitations in discriminant validity suggest substantial shared variance between dimensions.

**Discussion:**

Overall, these findings support the reliability and structural validity of the Spanish MIMI-16 and its use for assessing subtle forms of gender-based violence in organizational contexts, while highlighting the need for further research on the distinctiveness of its dimensions.

## 1 Introduction

In recent decades, research in social and organizational psychology has increasingly focused on subtle forms of gender-based discrimination embedded in everyday interactions and workplace contexts. Within this line of inquiry, gender microaggressions have been conceptualized as brief verbal, behavioral, or contextual exchanges that convey implicit messages of devaluation toward women, often in ambiguous ways and without explicit hostile intent (Sue et al., 2007; Sue, 2010), a characterization that resonates with earlier descriptions of *micromachismos* as low-intensity, socially legitimized practices of domination that operate through everyday interactions and tend to remain invisible due to their normalization (Bonino, 2004).

In organizational settings, repeated exposure to gender microaggressions directed at women has been linked to a range of adverse psychological and occupational outcomes, including increased emotional strain, lower well-being, and reduced professional self-confidence (Basford et al., 2014; Kim and Meister, 2023). Although these behaviors are typically characterized by their low intensity and high degree of normalization, their cumulative nature may contribute to the persistence of gender inequalities within workplace environments.

From a psychosocial perspective, gender microaggressions are not merely isolated incidents but interactional processes that may affect identity, perceived competence, and coping responses among women in professional contexts (Sue, 2010). Prior qualitative and theoretical work has emphasized that such experiences can elicit complex emotional reactions and require specific coping strategies, particularly when they occur repeatedly in environments marked by asymmetric power relations (Nadal and Haynes, 2012; Nadal et al., 2013).

Despite growing scholarly interest in this phenomenon, the systematic assessment of gender microaggressions in workplace settings remains limited, particularly outside Anglo-Saxon and Central European contexts. Most existing instruments were developed and validated within specific cultural frameworks, raising concerns about their structural validity and cross-cultural generalizability (Algner and Lorenz, 2022). Related measures—such as scales assessing hostile and benevolent sexism (Glick and Fiske, 1996, 2001) or instruments addressing symbolic violence in relational domains (Ferrer Pérez et al., 2008)—capture broader gender attitudes but do not assess the subtle, interactional nature of workplace microaggressions. In Spain, the *Escala de Micromachismos* (Torralba-Borrego and Garrido-Hernansaiz, 2021) has provided psychometric evidence for the measurement of *micromachista* attitudes in adult and university samples; however, it does not specifically target occupational interactions nor is it grounded in the microinsults–microinvalidations taxonomy proposed by Sue (2010).

Within this context, the Microinvalidations and Microinsults Scale (MIMI-16), developed by Algner and Lorenz (2022), represents one of the first instruments specifically designed to assess gender microaggressions directed at women in occupational settings, grounded in the taxonomy of microinvalidations and microinsults proposed by Sue (2010). Initial validation studies of this scale developed in German supported a correlated two-factor structure and provided evidence of adequate reliability and validity. However, the distinction between microinvalidations and microinsults remains theoretically and empirically complex. Given their conceptual proximity, these dimensions may exhibit substantial overlap, raising the possibility that gender microaggressions could also be represented as a more unified or hierarchically structured construct. Therefore, examining alternative factorial representations constitutes a relevant step in the validation of adapted versions of the instrument. Furthermore, particular attention should be given to their interrelationship, as they may share a substantial proportion of variance.

In addition, the expression, interpretation, and social normalization of microinvalidations and microinsults are likely to be shaped by cultural, linguistic, and organizational contexts. Accordingly, adaptation to a new linguistic setting requires empirical examination beyond simple translation, including the assessment of conceptual equivalence and structural validity in the target context.

Accordingly, the present study aims to adapt and validate the MIMI-16 in a sample of employed women in Spain. Specifically, we examine (a) the factorial structure of the instrument through confirmatory factor analysis (CFA), including the evaluation of theoretically plausible alternative models, (b) its internal consistency, (c) measurement invariance across two independent samples, and (d) multiple sources of validity evidence, including convergent and discriminant validity at the latent level, as well as criterion-related validity through associations with psychosocial risk and occupational health indicators. Following the methodological logic of the original validation study (Algner and Lorenz, 2022), analyses were conducted in two independent samples in order to assess the structural stability and replicability of the model in the Spanish occupational context.

## 2 Methods

### 2.1 Participants and procedure

The sample consisted of employed women (i.e., individuals who self-identified as women) from a wide range of occupational sectors and Spanish regions. A non-probabilistic sampling strategy was employed, aimed at maximizing occupational and territorial heterogeneity, as commonly recommended in psychometric validation studies to examine factorial stability and external validity in adapted instruments (Boateng et al., 2018; Worthington and Whittaker, 2006).

The inclusion criteria were: (a) self-identifying as a woman, (b) being 18 years of age or older, and (c) being actively working at the time of data collection. A total of 788 participants initially met the inclusion criteria. Cases with incomplete responses on the MIMI-16 were excluded using listwise deletion, resulting in the exclusion of 32 cases (Sample 1: *n* = 10; Sample 2: *n* = 22) and a final analytic sample of *N*_1_ = 352 and *N*_2_ = 404 participants. The proportion of excluded cases was low (approximately 4.1%), suggesting a limited impact on the representativeness of the analytic sample.

Data collection was conducted using an online self-administered questionnaire hosted on a secure digital platform. The study invitation was disseminated through collaborating organizations, professional networks, and social media channels. Participation was voluntary and anonymous, and all participants provided informed consent prior to completing the survey. Data were collected at two different time points for each sample, and recruitment was conducted through partially independent dissemination channels. No participant overlap between samples was allowed or detected.

#### 2.1.1 Sample 1

Sample 1 consisted of 352 women from diverse occupational sectors and Spanish regions. This sample was predominantly drawn from the public sector and was characterized by substantial work experience and broad territorial representation (see Table 1).

**Table 1:** Sociodemographic and occupational characteristics of sample 1 (*N* = 352).

Dimension	Category	%
Economic sector	Public administration	63.9
	Hospitality	17.9
	Financial and insurance activities	9.9
	Administrative and support services	6.8
	Wholesale and retail trade	1.4
Type of organization	Public sector	63.9
	Private sector	36.1
Main region	Basque country	19.0
	Valencian community	15.9
	Canary islands	12.8
	Andalusia	9.9
	Other provinces	42.4
Age	Under 20 years	7.1
	20–29 years	13.6
	30–39 years	12.2
	40–49 years	25.6
	50–60 years	32.4
	Over 60 years	9.1
Job tenure	<1 year	18.8
	Between 1 and 2 years	8.8
	Between 2 and 5 years	8.5
	Between 5 and 10 years	6.3
	>10 years	57.0
Nationality	Spanish	95.7
	Migrant person	4.3
Recognized disability	Yes	4.0
	No	96.0
Work modality	Full or partial telework	58.8
	Exclusively on-site work	41.2
Work organization	Solo work	40.6
	Stable team work	59.4
24/7 operational environment	Yes	13.1
	No	86.9
External violence (users, clients)	Has experienced work-related aggression or conflicts	14.5
	Has not experienced external violence	85.5

Overall, Sample 1 represents a heterogeneous and predominantly public-sector workforce, characterized by substantial professional experience, territorial diversity, and a notable presence of occupations involving public-facing roles and partial or full remote work.

#### 2.1.2 Sample 2

Sample 2 consisted of 404 women from a wide range of occupational sectors and Spanish regions. In contrast to Sample 1, this group exhibited a more heterogeneous profile in terms of geographic origin, age, and type of organization, with a greater representation of private-sector employment (see Table 2).

**Table 2:** Detailed sociodemographic and occupational characteristics of sample 2 (*N* = 404).

Dimension	Category	%
Economic sector	Public administration and defense	33.7
	Health and social services	28.2
	Information and communication	14.9
	Hospitality	13.9
	Commerce and distribution	7.4
	Construction	2.0
Type of organization	Private sector	75.2
	Public sector	24.8
Main region	Community of Madrid	51.0
	Galicia	16.8
	Basque country	7.9
	Cantabria	7.7
	Aragon	7.4
	Other regions	9.2
Age	Under 20 years	12.9
	20–29 years	19.6
	30–39 years	20.5
	40–49 years	20.0
	50–59 years	17.8
	Over 60 years	9.2
Job tenure	< 1 year	15.2
	Between 1 and 2 years	16.2
	Between 2 and 5 years	22.3
	Between 5 and 10 years	6.3
	>10 years	40
Nationality	Spanish	81.4
	Migrant person	18.6
Recognized disability	Yes	15.3
	No	84.7
Work modality	Full or partial telework	52.0
	Exclusively on-site work	48.0
24/7 operational environment	Yes	11.6
	No	88.4
External violence	Has experienced work-related external violence	18.3
	Has not experienced external violence	81.7

Overall, Sample 2 exhibited a younger and more diverse profile, with a stronger representation of the private sector, a higher prevalence of telework, and a substantial presence of participants from the healthcare and social services sectors. These differences enhance the comparative value of the study and allow for the examination of the stability of the measurement model across contrasting workplace contexts.

#### 2.1.3 Combined sample

In addition to analyses conducted separately in each sample, a complementary set of analyses was performed using the combined sample (*N* = 756), obtained by pooling Sample 1 and Sample 2. This strategy was adopted to examine the stability of the factorial structure and psychometric properties of the MIMI-16 in a large and diverse group of working women, while preserving the independence of the original samples for comparative purposes.

### 2.2 Instruments

We assessed (a) the target measure, the original MIMI-16 scale, and (b) a set of external criterion measures used to examine criterion-related and nomological validity evidence.

#### 2.2.1 Microinvalidations and Microinsults Scale (MIMI-16)

Gender microaggressions in the workplace were assessed using the Microinvalidations and Microinsults Scale (MIMI-16), developed by Algner and Lorenz (2022). The instrument consists of 16 items designed to measure two core dimensions of gender microaggressions: microinsults and microinvalidations. Microinsults refer to subtle comments or behaviors that implicitly question women’s professional competence, whereas microinvalidations involve the denial, minimization, or dismissal of experiences related to gender-based discrimination. Items are rated on a six-point rating-type scale, ranging from 1 (*strongly disagree*) to 6 (*strongly agree*), with higher scores indicating greater perceived exposure to gender microaggressions in the workplace.

The Spanish version of the MIMI-16 was developed following a translation and back-translation procedure, in accordance with established guidelines for cross-cultural adaptation of psychological instruments (Brislin, 1970; Beaton et al., 2000; Hambleton et al., 2005). In the first stage, two bilingual translators with experience in psychological research independently translated the original German version into Spanish. The resulting translations were then compared and synthesized into a single consensual version after resolving discrepancies. Subsequently, a third bilingual translator, independent from the initial translators, performed a back-translation into German to assess semantic and conceptual equivalence with the original scale.

Finally, the original authors of the MIMI-16 reviewed the Spanish version to ensure that each item preserved its intended meaning and conceptual content. Although no formal cognitive pretesting was conducted, this step is acknowledged as a limitation. The resulting version was considered suitable for empirical evaluation in the Spanish workplace context. The German original and the Spanish translation of the MIMI-16 items are presented in Table 3.

**Table 3:** MIMI 16 German version and Spanish translation.

Item		German original MIMI-16	Spanish version MIMI-16
Invalidations	1	Es kommt vor, dass männliche kollegen ein meeting fortsetzen, nachdem die frauen den raum verlassen haben	Mis compañeros varones continúan las reuniones una vez las mujeres hemos salido de la habitación
Invalidations	2	Ich habe das gefühl, dass man mir weniger zutraut, weil ich eine frau bin	Tengo la sensación de que la gente espera menos de mí porque soy mujer
Invalidations	3	Frauen bekommen komplimente für ihr äußeres, männer für ihre arbeitsleistung	Las mujeres reciben cumplidos por su apariencia, los hombres por su desempeño laboral
Invalidations	4	Andere nehmen an, dass sich familiengründung negativ auf die arbeitsleistung von frauen auswirkt	La gente asume que formar una familia tiene un impacto negativo en el desempeño laboral de las mujeres
Invalidations	5	Meine durchsetzungskraft wird im beruflichen kontext negativ bewertet	Mi asertividad es vista negativamente en un contexto profesional
Invalidations	6	Man hat mir schon einmal zu verstehen gegeben, dass meine berufliche leistung anders bewertet wird, als die von männern	Me han dicho que mi desempeño profesional se evalúa de manera diferente a la de los hombres
Invalidations	7	Ich habe das gefühl ständig meine berufliche qualifikation beweisen zu müssen	Tengo la sensación de que tengo que demostrar desempeño laboral todo el tiempo
Invalidations	8	Vorschläge werden eher akzeptiert, wenn sie von einem mann geäußert werden	Es más probable que se acepten las sugerencias si las hace un hombre
Insults	9	Manchmal bekomme ich komplimente, die ich als unangebracht empfinde	A veces recibo cumplidos que considero inapropiados
Insults	10	Unter meinen kolleg*innen werden manchmal anzügliche witze gegenüber frauen gemacht	Mis compañeros varones hacen bromas lascivas sobre mujeres
Insults	11	Es ist schon vorgekommen, dass kolleg*innen meine kleidung kommentierten	Mis compañeros han comentado mi forma de vestir
Insults	12	Ich wurde in meinem arbeitsumfeld schon nach meinem menstruationszyklus gefragt	Han hecho comentarios sobre mi ciclo menstrual en mi lugar de trabajo
Insults	13	Ich bin im beruflichen kontext sexualisiert worden	He sido sexualizada en un contexto profesional
Insults	14	Es kam schon vor, dass man mir an meinem arbeitsplatz anzügliche kosenamen gegeben hat	En mi lugar de trabajo me han puesto motes sugerentes
Insults	15	Mein verhalten wurde schon einmal aufgrund meines geschlechts scherzhaft nachgeahmt	Mi comportamiento ha sido imitado en broma debido a mi género
Insults	16	Ich habe das gefühl, dass mein aussehen mehr für meinen beruflichen erfolg verantwortlich ist, als meine qualifikation	Siento que mi apariencia es más responsable de mi éxito profesional que mi desempeño laboral

Translation–back-translation with expert review.

### 2.3 Criterion measures

To examine criterion-related and nomological validity evidence, the MIMI-16 was correlated with a set of psychological and organizational variables that have been theoretically and empirically linked to experiences of gender microaggressions in the workplace.

#### 2.3.1 Psychological distress

Psychological distress was assessed using the Spanish version of the 12-item General Health Questionnaire (GHQ-12; Goldberg and Williams, 1988), validated in the Spanish population by Sánchez-López and Dresch (2008). Items are rated on a 4-point rating scale ranging from 0 (*less than usual*) to 3 (*much more than usual*). An example item is: “Have you felt constantly under strain?”. Total scores were computed, with higher scores indicating greater psychological distress.

#### 2.3.2 Burnout

Burnout was assessed using the Spanish version of the Maslach Burnout Inventory (MBI; Maslach et al., 1996). The MBI conceptualizes burnout as a multidimensional construct comprising emotional exhaustion, depersonalization, and reduced personal accomplishment. Items are rated on a 7-point frequency scale ranging from 0 (*never*) to 6 (*every day*). Example items include: “I feel emotionally exhausted by my work” (emotional exhaustion), “I have become more callous toward people since I took this job” (depersonalization), and “I feel I am positively influencing other people’s lives through my work” (personal accomplishment). The Spanish version of the MBI has demonstrated adequate reliability and construct validity in occupational samples (e.g., Moreno-Jiménez et al., 2001; Salanova et al., 2000).

#### 2.3.3 Work engagement

Work engagement was assessed using the Spanish version of the 9-item Utrecht Work Engagement Scale (UWES-9; Schaufeli et al., 2006). The scale measures three dimensions of engagement—vigor, dedication, and absorption—reflecting a positive, work-related affective–motivational state. Example items include: “At my work, I feel bursting with energy” (vigor), “I am enthusiastic about my job” (dedication), and “I am immersed in my work” (absorption). Items are rated on a 7-point frequency scale ranging from 0 (*never*) to 6 (*every day*). Evidence from Spanish samples included in the cross-national validation study supports the reliability and factorial validity of the UWES-9 (Schaufeli et al., 2006).

#### 2.3.4 Job satisfaction

Job satisfaction was assessed using the 5-item Brief Job Satisfaction Measure originally developed by Judge et al. (1998). For the present study, we used the Spanish adaptation of this brief scale developed by Extremera et al. (2018). The instrument assesses individuals’ global affective evaluation of their job. Items are rated on a 7-point rating scale ranging from 1 (*strongly disagree*) to 7 (*strongly agree*). An example item is: “I feel fairly satisfied with my present job.” Higher mean scores indicate greater overall job satisfaction.

#### 2.3.5 Core self-evaluations (CSE)

Core self-evaluations were assessed using the Spanish version of the Core Self-Evaluations Scale (CSES; Judge et al., 2003), validated by Beléndez et al. (2018). The CSES is a 12-item measure of a broad dispositional construct encompassing self-esteem, generalized self-efficacy, locus of control, and emotional stability. Items are rated on a 5-point rating scale ranging from 1 (*strongly disagree*) to 5 (*strongly agree*). An example item is: “I am confident in my abilities.” Higher scores indicate more positive core self-evaluations.

#### 2.3.6 Occupational self-efficacy

Occupational self-efficacy was assessed using the short version of the Spanish version of the Occupational Self-Efficacy Scale (Rigotti et al., 2008). The scale consists of six items measuring individuals’ confidence in their ability to successfully deal with work-related demands and problems. Items are rated on a 6-point rating scale ranging from 1 (*not at all true*) to 6 (*completely true*). An example item is: “I can effectively solve the problems that arise in my work.” Higher scores indicate greater perceived occupational self-efficacy.

#### 2.3.7 Turnover intention (TIS)

Turnover intention was assessed using the 6-item Turnover Intention Scale (TIS-6), originally developed by Roodt (2004) and later validated by Bothma and Roodt (2013). For the present study, we used the Spanish-language version of the scale (Morales Hernández, 2021). The scale consists of six items assessing the frequency with which individuals consider leaving their current job. Responses are recorded on a 5-point rating scale ranging from 1 (*never*) to 5 (*very often*). An example item is: “How often have you thought about leaving your current job?” Higher scores indicate stronger turnover intentions.

#### 2.3.8 Workplace social relations and exposure to interpersonal conflict

Workplace social relations and exposure to interpersonal conflict were assessed using selected items from the FPSICO 4.1 psychosocial risk questionnaire (NTP 926), developed by the Spanish National Institute for Safety and Health at Work (INSST) and widely used in Spain to evaluate psychosocial working conditions. Specifically, items from the Social Relations and Support factor assessing problematic interpersonal interactions and exposure to workplace violence were selected. Items are rated on a 5-point rating-type scale ranging from 0 (*never*) to 4 (*always*). An example item is: “Do conflicts or tense situations frequently arise in your work environment?” In addition, a composite index of social relations and support was computed by averaging the selected items, with higher scores indicating poorer social relations and greater exposure to adverse interpersonal experiences at work.

### 2.4 Data analysis

Preliminary data screening and assumption checking—including the examination of missing values, univariate normality, skewness, and kurtosis—were conducted using IBM SPSS Statistics (Version 27). Missing data were handled using listwise deletion, because only participants who completed all MIMI-16 items were retained for analysis. Therefore, CFA and multigroup CFA models were estimated using complete cases, and no imputation procedures were applied. CFA were estimated using the robust weighted least squares estimator with mean and variance adjustment (WLSMV), which is recommended for ordinal indicators. Given the six-point rating-type response format of the MIMI-16 and the observed deviations from normality in several items, WLSMV provides more appropriate parameter estimates and standard errors than maximum likelihood estimators. Although the original study employed MLR, the present study adopted WLSMV to better accommodate the ordinal nature of the data.

The model was first estimated using the combined sample (*N* = 756) and subsequently examined in two independent subsamples (*N* = 352 and *N* = 404) to assess the stability of the factorial structure across samples. In addition to the hypothesized correlated two-factor model, alternative theoretically plausible models (i.e., unidimensional, higher-order, and bifactor models) were also estimated and compared to evaluate the adequacy of the factorial structure. Model fit was evaluated using multiple indices: the chi-square statistic (*χ*^2^), the Comparative Fit Index (CFI), the Tucker–Lewis Index (TLI), the Root Mean Square Error of Approximation (RMSEA) with 90% confidence intervals, and the Standardized Root Mean Square Residual (SRMR), following commonly accepted criteria (Hu and Bentler, 1999).

Given that the MIMI-16 has a clearly specified and empirically supported factorial structure in its original version (Algner and Lorenz, 2022), the present study adopted a confirmatory approach. Accordingly, no exploratory factor analysis was conducted, as the aim was not to identify an unknown latent structure but to evaluate the replicability and stability of a previously specified theoretical model in the Spanish workplace context. This decision is consistent with current methodological recommendations, which discourage the use of exploratory analyses when strong theoretical and empirical foundations exist and instead prioritize confirmatory methods for testing validated models (Boateng et al., 2018; Flora and Flake, 2017; Worthington and Whittaker, 2006).

Standardized factor loadings were computed, and internal consistency was assessed using Cronbach’s alpha (*α*), McDonald’s omega (*ω*), and composite reliability (CR). Evidence of convergent and discriminant validity was examined through the average variance extracted (AVE) and the Fornell–Larcker criterion (Fornell and Larcker, 1981). Additionally, the heterotrait–monotrait ratio (HTMT) was computed as a complementary indicator of discriminant validity. These analyses were conducted using the semTools package (Jorgensen et al., 2021) and the MBESS package in R.

Finally, the results of the original validation study by Algner and Lorenz (2022) were used as a comparative benchmark for interpreting model fit and psychometric performance.

In addition to Pearson bivariate correlations used to examine criterion-related validity at the scale level, supplementary Spearman rank-order correlations were computed to assess the robustness of the observed associations given the ordinal nature of the response formats. This sensitivity analysis allowed us to verify that the pattern of relationships did not depend on parametric assumptions.

## 3 Results

### 3.1 Descriptive statistics and correlation analyses

Item-level descriptive statistics for the MIMI-16 are presented in Table 4. Across both samples, most items showed pronounced positive skewness and elevated kurtosis, indicating a strong concentration of responses at the lower end of the scale. Several items, particularly those reflecting more explicit forms of microinsults, exhibited substantial departures from normality, consistent with the presence of floor effects.

**Table 4:** Item-level descriptive statistics of the MIMI-16 by sample.

Item	Mean S1	SD S1	Skew S1	Kurt S1	Mean S2	SD S2	Skew S2	Kurt S2
mi_1	1.54	1.28	2.44	4.81	1.26	0.90	3.81	14.39
mi_2	1.47	1.18	2.68	6.36	1.32	0.92	3.22	10.39
mi_3	1.76	1.38	1.74	1.86	1.58	1.24	2.26	4.25
mi_4	2.35	1.81	1.03	−0.43	2.27	1.67	1.04	−0.24
mi_5	1.98	1.51	1.41	0.79	1.77	1.39	1.80	2.13
mi_6	1.70	1.39	2.04	3.01	1.54	1.26	2.41	4.77
mi_7	2.54	1.92	0.79	−0.99	2.52	1.86	0.79	−0.92
mi_8	1.94	1.56	1.53	1.05	1.67	1.41	2.12	3.24
mi_9	1.65	1.26	2.16	4.00	1.36	0.96	3.10	9.53
mi_10	1.59	1.24	2.39	4.98	1.34	0.98	3.53	12.57
mi_11	1.46	1.09	2.66	6.68	1.28	0.87	3.70	14.12
mi_12	1.20	0.69	4.53	23.42	1.12	0.59	5.91	39.04
mi_13	1.30	0.88	3.44	12.14	1.15	0.66	5.33	30.91
mi_14	1.20	0.73	4.48	21.79	1.08	0.49	6.94	52.86
mi_15	1.23	0.82	4.37	20.13	1.12	0.60	5.81	37.04
mi_16	1.55	1.24	2.47	5.26	1.28	0.79	3.51	13.64

S1, Sample 1; S2, Sample 2; Skew, skewness; Kurt, kurtosis.

Following the methodological approach adopted in the original validation study of the MIMI-16 (Algner and Lorenz, 2022), Pearson bivariate correlations were first computed to examine the pattern of associations between MIMI-16 scores and the criterion variables related to psychosocial functioning and organizational outcomes.

Although the MIMI-16 items are ordinal, correlations were computed on aggregated scale scores, which approximate continuous distributions. To ensure robustness of the findings, supplementary analyses using Spearman rank-order correlations were also conducted. These yielded an equivalent pattern of associations in terms of direction, magnitude, and statistical significance across both samples (see Supplementary Tables S1, S2), supporting the stability of the results.

As an external organizational indicator, a global index of social relations and support was included. This index was derived from the FPSICO 4.1 psychosocial risk assessment instrument and computed by aggregating items assessing social relations, support, and exposure to problematic interpersonal interactions at work (see notes to Tables 5, 6). Additional correlations were examined with indicators of psychological health and work-related psychosocial variables. For Sample 1, these included psychological distress, work engagement, job satisfaction, and burnout (emotional exhaustion, depersonalization, and personal accomplishment). For Sample 2, analyses focused on core self-evaluations, turnover intention, and general self-efficacy.

**Table 5:** Pearson correlations between the MIMI-16 and psychosocial and organizational variables (sample 1).

Scale and subscales	MIMI-16 total	Microinsults	Microinvalidations	Social relations and support	Work engagement	Psychological distress (GHQ)	Job satisfaction	Emotional exhaustion	Depersonalization	Personal accomplishment
MIMI total	1	0.848**	0.936**	0.551**	−0.312**	0.374**	−0.415**	0.398**	0.437**	0.117*
Microinsults	0.848**	1	0.606**	0.495**	−0.281**	0.290**	−0.384**	0.272**	0.302**	0.149**
Microinvalidations	0.936**	0.606**	1	0.497**	−0.280**	0.369**	−0.366**	0.415**	0.454**	0.076

*p < 0.05; **p < 0.01.

**Table 6:** Pearson correlations between the MIMI-16 and psychosocial and organizational variables (sample 2).

Scale and subscales	MIMI-16 total	Microinsults	Microinvalidations	Social relations and support	Core self-evaluations (CSE)	Turnover intention (TIS)	Occupational self-efficacy
MIMI total	1	0.841**	0.951**	0.343**	−0.222**	0.151**	−0.117**
Microinsults	0.841**	1	0.631**	0.291**	−0.139**	0.102*	−0.127*
Microinvalidations	0.951**	0.631**	1	0.325**	−0.238**	0.158**	−0.095

*p < 0.05; **p < 0.01.

#### 3.1.1 Pearson correlations (sample 1)

In Sample 1, the total MIMI-16 score showed a moderate to strong positive association with the global index of social relations and support (*r* = 0.551, *p* < 0.01), psychological distress (GHQ total; *r* = 0.374, *p* < 0.01), emotional exhaustion (*r* = 0.398, *p* < 0.01), and depersonalization (*r* = 0.437, *p* < 0.01). In contrast, negative associations were observed with work engagement (*r* = −0.312, *p* < 0.01) and job satisfaction (*r* = −0.415, *p* < 0.01). The association with personal accomplishment was smaller in magnitude (*r* = 0.117, *p* < 0.05).

When the two dimensions of the MIMI-16 were examined separately, microinsults and microinvalidations exhibited associations in the same direction and of comparable magnitude. Both dimensions were statistically significantly correlated with the global index of social relations and support and with indicators of psychological health and work-related well-being.

Overall, these results indicate that higher levels of perceived gender microaggressions are consistently associated with poorer psychosocial functioning and less favorable work-related outcomes, with stronger associations observed for proximal indicators such as burnout, distress, and job satisfaction. The full set of correlation coefficients for Sample 1 is reported in Table 5.

#### 3.1.2 Pearson correlations (sample 2)

In Sample 2, the total MIMI-16 score showed a positive association with the global index of social relations and support (*r* = 0.343, *p* < 0.01) and with turnover intention (*r* = 0.151, *p* < 0.01), as well as negative associations with core self-evaluations (CSE total; *r* = −0.222, *p* < 0.01) and occupational self-efficacy (*r* = −0.117, *p* < 0.01).

Consistent with the findings for Sample 1, the microinsults and microinvalidations dimensions exhibited associations in the same direction and of comparable magnitude. The full set of correlation coefficients for Sample 2 is presented in Table 6.

### 3.2 Confirmatory factor analysis

Following the original two-factor structure proposed by Algner and Lorenz (2022), a correlated two-factor model was specified, comprising microinvalidations (mi_1–mi_8) and microinsults (mi_9–mi_16). Given the ordinal nature of the response scale and the marked skewness observed in several items, models were estimated using WLSMV.

The model was first tested separately in each sample and subsequently in the pooled dataset. Fit indices for the correlated two-factor model across samples are presented in Table 7. In Sample 1 (*N* = 352), the model showed overall acceptable fit, although RMSEA values were slightly above conventional thresholds. In Sample 2 (*N* = 404), fit indices were good or very good. In the combined sample (*N* = 756), the model demonstrated excellent overall fit, *χ*^2^ (103) = 223.09, *p* < 0.001, CFI = 0.983, TLI = 0.981, SRMR = 0.057, and RMSEA = 0.051, 90% CI [0.045, 0.057].

**Table 7:** CFA fit indices (sample 1, sample 2, total).

Sample	*N*	*χ* ^2^	d*f*	*p*	CFI	TLI	SRMR	RMSEA	90% CI RMSEA
Sample 1	352	260.71	103	<0.001	0.977	0.973	0.071	0.066	[0.056, 0.076]
Sample 2	404	171.74	103	<0.001	0.986	0.984	0.064	0.041	[0.030, 0.051]
Total sample	756	223.09	103	<0.001	0.983	0.981	0.057	0.051	[0.045, 0.057]

Estimation method, WLSMV with ordinal indicators. Reported values correspond to scaled fit indices.

To further evaluate the dimensional structure of the instrument, several theoretically plausible alternative models (i.e., unidimensional, higher-order, and bifactor models) were estimated and compared. The corresponding fit indices are presented in Table 8. Comparative analyses indicated that the unidimensional model provided inferior fit, whereas the higher-order model did not yield interpretable parameter estimates. Although the bifactor model showed improved nominal fit, it resulted in inadmissible solutions (negative latent variances), suggesting model instability and potential overfitting.

**Table 8:** Comparison of alternative factorial models for the MIMI-16.

Model	*χ* ^2^	d*f*	CFI	TLI	RMSEA	90% CI RMSEA	SRMR	Notes
Unidimensional	609.28	104	0.96	0.95	0.080	[074, 0.086]	0.087	Poorer fit
Two correlated factors	341.07	103	0.98	0.98	0.055	[049, 0.062]	0.059	Retained as most interpretable solution
Higher-order	—	102	—	—	—	—	0.059	Did not yield interpretable fit indices
Bifactor	184.30	88	0.99	0.99	0.038	[0.030, 0.046]	0.034	Inadmissible solution (negative latent variances)

Taken together, these results support the correlated two-factor model as the most parsimonious and interpretable solution, while acknowledging the substantial shared variance between factors.

#### 3.2.1 Standardized factor loadings

Standardized loadings, presented in Table 9, in the pooled dataset ranged from 0.65 to 0.93 for microinvalidations and from 0.80 to 0.91 for microinsults, indicating strong associations between observed indicators and their respective latent factors.

**Table 9:** Standardized factor loadings.

Item	Factor	*λ* (standardized)
mi_1	Microinvalidations	0.73
mi_2	Microinvalidations	0.88
mi_3	Microinvalidations	0.86
mi_4	Microinvalidations	0.72
mi_5	Microinvalidations	0.80
mi_6	Microinvalidations	0.92
mi_7	Microinvalidations	0.71
mi_8	Microinvalidations	0.89
mi_9	Microinsults	0.84
mi_10	Microinsults	0.82
mi_11	Microinsults	0.80
mi_12	Microinsults	0.88
mi_13	Microinsults	0.91
mi_14	Microinsults	0.83
mi_15	Microinsults	0.89
mi_16	Microinsults	0.81

All loadings *p* < 0.001.

All items loaded substantially on their intended factors, supporting the internal coherence of the two dimensions. However, consistent with the high correlation observed between factors, these results also suggest that both dimensions capture closely related aspects of workplace gender microaggressions.

### 3.3 Measurement invariance across samples

To examine the stability of the MIMI-16 measurement model across independent samples of working women, multigroup CFA were conducted using the WLSMV estimator, which is recommended for ordinal indicators. The results are presented in Table 10.

**Table 10:** Measurement invariance across samples (WLSMV estimation).

Model	CFI	RMSEA	SRMR	ΔCFI	ΔRMSEA	ΔSRMR
Configural	0.984	0.048	0.065	—	—	—
Metric	0.987	0.043	0.075	+0.003	−0.005	+0.010
Scalar	0.984	0.046	0.065	−0.003	+0.003	−0.010

Δ values are relative to the preceding model.

Measurement invariance was tested sequentially following standard procedures for ordinal data: configural invariance (same factorial structure across groups), metric invariance (equal factor loadings), and scalar invariance (equal factor loadings and thresholds). Because WLSMV estimation does not rely on traditional *χ*^2^ difference testing, decisions were based on changes in approximate fit indices, in line with current methodological recommendations (Cheung and Rensvold, 2002; Chen, 2007). Specifically, invariance was supported when changes in CFI were ≤0.010, RMSEA ≤0.015, and SRMR ≤0.030 for metric and ≤0.010 for scalar invariance.

The configural model demonstrated acceptable fit (CFI = 0.984; RMSEA = 0.048; SRMR = 0.065), indicating that the two-factor structure was similarly represented in both samples. Imposing equality constraints on factor loadings (metric invariance) did not result in a meaningful deterioration of model fit (CFI = 0.987; RMSEA = 0.043; SRMR = 0.075), with changes in fit indices (ΔCFI = 0.003; ΔRMSEA = −0.005; ΔSRMR = 0.010) well below recommended thresholds. Likewise, the scalar invariance model (equal loadings and thresholds) showed comparable fit (CFI = 0.984; RMSEA = 0.046; SRMR = 0.065), again meeting established criteria for invariance.

Taken together, these findings support configural, metric, and scalar invariance of the MIMI-16 across samples, indicating that the factorial structure, factor loadings, and item thresholds are largely comparable. This level of invariance supports comparisons of structural relations across samples. In the present study, scalar invariance was primarily established to confirm measurement stability rather than to support latent mean comparisons.

### 3.4 Reliability and latent convergent/discriminant validity

Internal consistency was high for both dimensions. Ordinal alpha values were 0.92 (microinvalidations) and 0.95 (microinsults). Model-based omega coefficients were 0.88 and 0.89, respectively. Composite reliability (CR) values exceeded 0.93 for both factors, indicating strong internal consistency.

Convergent validity at the latent level was examined through the average variance extracted (AVE). AVE values were 0.64 for microinvalidations and 0.73 for microinsults, exceeding the recommended 0.50 threshold and suggesting adequate to strong convergence between indicators and their respective latent constructs.

The latent correlation between factors was high (*r* ≈ 0.83), reflecting the conceptual proximity of both dimensions. Discriminant validity was evaluated using the Fornell–Larcker criterion. Specifically, the AVE for microinvalidations (0.64) was lower than the squared inter-factor correlation (*r*^2^ ≈ 0.69), indicating that the criterion was not met for this dimension. To complement this analysis, the heterotrait–monotrait ratio (HTMT) was computed, yielding a value of 0.83, which falls below the recommended threshold of 0.85 and provides additional support for discriminant validity. The results are presented in Table 11.

**Table 11:** Reliability, AVE/CR and discriminant validity for the MIMI-16.

Factor	*α*	Ordinal *α*	*ω*	CR	AVE	*r*	*r* ^2^	Discriminant validity
Microinvalidations	0.85	0.92	0.88	0.93	0.64	0.83	0.69	Partial
Microinsults	0.86	0.95	0.89	0.95	0.73	0.83	0.69	Adequate

Taken together, these results indicate substantial shared variance between microinvalidations and microinsults. However, considering the HTMT results and the theoretical relatedness of both dimensions within the broader microaggression framework, the distinction between factors remains interpretable, albeit with caution.

Beyond latent-level evidence, the pattern of associations with external psychosocial and organizational variables provides criterion-related and nomological validity evidence, rather than convergent or discriminant validity in the strict psychometric sense.

### 3.5 Comparison of factorial model fit between Spanish and German samples

To contextualize the findings, descriptive comparisons were conducted between the Spanish and German validation studies. Across datasets, global fit indices were broadly comparable, with Spanish samples showing CFI and RMSEA values within—and in some cases exceeding—those reported in the original German validation. The results are presented in Table 12.

**Table 12:** Comparison Spanish vs.German fit indices.

Sample	*N*	*χ* ^2^	d*f*	CFI	TLI	SRMR	RMSEA	90% CI RMSEA
German-study 1 (dataset 2)	250	150.34	103	0.959	0.953	0.049	0.046	[0.029, 0.062]
German-study 2 (dataset 1)	306	207.43	103	0.936	0.926	0.049	0.062	[0.050, 0.074]
German-study 2 (dataset 2)	306	174.84	103	0.960	0.954	0.042	0.051	[0.038, 0.064]
Spanish-sample 1	352	260.71	103	0.977	0.973	0.071	0.066	[0.056, 0.076]
Spanish-sample 2	404	171.74	103	0.986	0.984	0.064	0.041	[0.030, 0.051]
Spanish-total sample	756	223.09	103	0.983	0.981	0.057	0.051	[0.045, 0.057]

Spanish models estimated using WLSMV with ordinal indicators.

These comparisons should be interpreted with caution, as they are descriptive in nature and do not reflect formal statistical equivalence testing across samples or studies.

Nevertheless, the overall similarity in model fit provides preliminary support for the structural robustness of the MIMI-16 across different linguistic and cultural contexts.

## 4 Discussion

The present study sought to examine the factorial structure and measurement invariance of the Spanish adaptation of the MIMI-16 in two independent samples of working women. Overall, the results supported the replication of the original correlated two-factor model and provided consistent evidence of structural validity across samples.

However, the substantial correlation observed between microinvalidations and microinsults, together with the partial limitations identified in discriminant validity, points to a considerable degree of shared variance between both dimensions. From this perspective, the findings are more appropriately interpreted as reflecting a two-dimensional structure comprising closely related facets of a broader underlying construct, rather than fully independent factors.

This interpretation is consistent with theoretical accounts of microaggressions as a unified phenomenon expressed through distinct but interrelated forms (Sue, 2010), in which microinvalidations and microinsults represent differentiated yet overlapping manifestations of symbolic gender-based devaluation.

Notably, alternative factorial representations—including unidimensional, higher-order, and bifactor models—were examined but either showed inferior fit or yielded inadmissible or unstable solutions. Taken together, model comparisons indicate that although a general factor may underlie gender microaggressions, the correlated two-factor structure provides the most stable and interpretable representation of the construct in the Spanish context.

### 4.1 Factorial structure and psychometric properties

Model fit across both independent samples and the pooled dataset supported the adequacy of the correlated two-factor structure under WLSMV estimation. Standardized factor loadings were generally moderate to high, indicating that items functioned consistently as indicators of their respective dimensions. Microinvalidations showed slightly greater variability in loadings than microinsults, which may reflect the broader and more ambiguous nature of invalidating experiences in workplace interactions. This pattern is consistent with theoretical accounts describing microinvalidations as subtle, often normalized practices that may be differentially interpreted depending on organizational culture and role expectations (Sue, 2010; Nadal and Haynes, 2012).

With respect to reliability, both dimensions demonstrated high internal consistency, as indicated by Cronbach’s alpha, McDonald’s omega, and ordinal alpha coefficients. Composite reliability and average variance extracted values further supported the convergent validity of the scale. However, the AVE for microinvalidations was lower than the squared inter-factor correlation, indicating that the Fornell–Larcker criterion was not met for this dimension. This result reflects the substantial empirical overlap between microinvalidations and microinsults, which was further qualified by HTMT values below recommended thresholds, supporting a cautious but defensible distinction between factors. In line with these findings, alternative factorial representations (unidimensional, higher-order, and bifactor models) were examined but either showed inferior fit or yielded inadmissible or unstable solutions.

Taken together, these results suggest that the two dimensions capture differentiated yet highly interrelated manifestations of gender microaggressions. This pattern is consistent with theoretical perspectives that conceptualize microaggressions as a unified phenomenon expressed through distinct but overlapping forms (Sue, 2010), thereby supporting the retention of a correlated two-factor structure despite the high degree of shared variance.

Importantly, multigroup analyses supported configural, metric, and scalar invariance across samples, indicating that the factorial structure, loadings, and thresholds operated comparably in both subsamples. This strengthens confidence in the structural stability of the instrument and supports the comparability of latent scores across independent samples within the same cultural context.

### 4.2 Criterion-related validity and nomological evidence

Beyond internal structure, the pattern of associations between MIMI-16 scores and external criteria provided further support for construct validity. Higher exposure to gender microaggressions was consistently associated with poorer workplace social relations, greater psychological distress and burnout, lower work engagement and job satisfaction, and higher turnover intention.

Importantly, the strength of these associations differed across samples, with stronger relationships observed in Sample 1, which included more proximal, state-like indicators (e.g., burnout, distress, job satisfaction), and weaker associations in Sample 2, which relied on more distal and dispositional constructs (e.g., core self-evaluations and occupational self-efficacy).

This pattern was robust to alternative (nonparametric) correlation estimates, with supplementary analyses yielding an equivalent pattern of results across both independent samples. These findings align with prior research documenting the cumulative psychosocial costs of gender microaggressions in organizational settings (Basford et al., 2014; Kim and Meister, 2023) and reinforce the scale’s nomological validity within occupational research contexts.

Taken together, the present findings provide converging evidence supporting the reliability, structural validity, measurement invariance, and criterion-related validity of the Spanish version of the MIMI-16. While some psychometric indicators suggest substantial overlap between dimensions, the overall pattern of results supports its use as a theoretically grounded measure of workplace gender microaggressions in both research and applied contexts.

### 4.3 Limitations and future research

Several limitations of the present study should be acknowledged. First, the use of a non-probabilistic sampling strategy limits the generalizability of the findings beyond the populations and occupational contexts represented in the study. Although efforts were made to maximize heterogeneity in terms of sectors, regions, and employment conditions, future research should replicate these findings using probabilistic or stratified sampling designs.

Second, the cross-sectional design precludes causal inferences regarding the relationships between gender microaggressions and psychosocial or occupational outcomes. Longitudinal designs would be particularly valuable for examining the temporal dynamics and cumulative effects of workplace microaggressions, as well as their potential role in the development of psychological distress, burnout, and turnover intention over time.

Third, all variables were assessed using self-report measures collected within the same survey context, which may raise concerns regarding common-method variance. Although the primary aim of the present study was the psychometric validation of a measurement instrument rather than causal modeling, and self-report methodologies are standard in scale validation research, future studies should consider incorporating multi-source assessments (e.g., supervisor ratings or objective organizational indicators) or longitudinal designs to further examine the robustness of the observed associations. Nevertheless, the replication of the expected pattern of associations across two independent samples reduces the likelihood that the findings are solely attributable to shared method variance.

Fourth, although the scale demonstrated acceptable levels of discriminant validity according to HTMT criteria, the high shared variance between microinvalidations and microinsults suggests that their distinction should be interpreted with caution. This overlap may reflect partially overlapping manifestations of gender microaggressions rather than fully distinct constructs, and future research should further examine the dimensional structure of the instrument using alternative modeling approaches and diverse samples.

Fifth, the study did not include established Spanish-language measures assessing conceptually related constructs (e.g., micromachismos or ambivalent sexism), which limited the extent to which convergent validity could be examined at the construct level. Incorporating such measures in future studies would strengthen convergent validity evidence.

Sixth, different sets of criterion variables were used across samples, including more proximal, state-like outcomes in Sample 1 and more distal, dispositional constructs in Sample 2. Although this design allowed us to examine the scale across diverse nomological contexts, it limited the direct comparability of criterion-related validity evidence between samples. Future studies should include overlapping criterion measures across samples to enhance comparability.

Finally, although the MIMI-16 was originally developed and validated in Germany, its application in different cultural contexts requires caution. When using the scale in other national settings, future research should consider that the specific manifestations of gender microaggressions may vary as a function of cultural and organizational context. Although the present study validated a Spanish-language version of the MIMI-16, data were collected exclusively in Spain; therefore, further research should adapt and examine the measure in other Spanish-speaking cultural contexts.

These findings should be interpreted within the constraints of cross-sectional self-report data and require replication across different cultural and organizational contexts.

## 5 Conclusion

In conclusion, the present study provides initial empirical support for the reliability and validity of the Spanish version of the MIMI-16 as an instrument for assessing gender microaggressions in occupational settings. The original correlated two-factor structure—comprising microinvalidations and microinsults—was replicated across two independent samples and demonstrated structural stability across groups, in line with the specification proposed by Algner and Lorenz (2022).

From a psychometric standpoint, the scale demonstrated adequate factor loadings, high levels of internal consistency, and evidence of convergent validity. At the same time, the strong correlation between dimensions and partial limitations in discriminant validity indicate that both factors capture closely related aspects of a broader underlying construct. This pattern is consistent with theoretical models of microaggressions (Sue, 2010; Nadal and Haynes, 2012), which conceptualize these phenomena as interconnected expressions of gender-based devaluation rather than fully independent categories.

Overall, the findings support the use of the MIMI-16 as a theoretically grounded and psychometrically adequate tool for examining subtle forms of gender-based discrimination in workplace contexts, while highlighting the need for continued research to further refine its dimensional structure and cross-cultural applicability.

## Data Availability

The dataset contains sensitive information and will be made available by the authors upon reasonable request, in accordance with ethical and confidentiality requirements.
