# Supplementary material for: Measuring workplace gender microaggressions in Spain: validation and measurement invariance of the Spanish version of the MIMI-16
Source: Front Psychol. 2026 May 8;17:1821085. doi: 10.3389/fpsyg.2026.1821085 (PMC13194036; doi:10.3389/fpsyg.2026.1821085)
Supplement: Supplementary file 1 [file Table_1.docx]

Supplementary Material

*Supplementary Table S1. Spearman Rank-Order Correlations Between the MIMI-16 and Psychosocial and Organizational Variables (Sample 1)*

|  | MIMI-16 Total | | Microinsults | | | Microinvalidations | | Social Relations and Support | | Work Engagement | | Psychological Distress | Job Satisfaction | | Emotional Exhaustion | | | Depersonalization | | Personal Accomplishment | | |
| --- | --- | --- | --- | --- | --- | --- | --- | --- | --- | --- | --- | --- | --- | --- | --- | --- | --- | --- | --- | --- | --- | --- |
| MIMI Total | | 1.000 | | .822^**^ | .957^**^ | | .506^**^ | | -.350^**^ | | .377^**^ | | | -.385^**^ | | .425^**^ | | | ,456^**^ | | ,162^**^ |  |
| Microinsults | | .822^**^ | | 1.000 | .669^**^ | | .420^**^ | | -.331^**^ | | .278^**^ | | | -.425^**^ | | .295^**^ | | | ,343^**^ | | ,116^*^ |  |
| Microinvalidations | | .957^**^ | | .669^**^ | 1.000 | | .476^**^ | | -.310^**^ | | .396^**^ | | | -.340^**^ | | .446^**^ | | | ,454^**^ | | ,131^*^ |  |
| *Note*: * p < .05. ** p < .01. | | | | | | | | | | | | | | | | |  |  |  |  |  |  |

*Supplementary Table S2. Spearman Rank-Order Correlations Between the MIMI-16 and Psychosocial and Organizational Variables (Sample 2)*

|  | MIMI-16 Total | | Microinsults | | Microinvalidations | | Social Relations and Support | | Core Self-Evaluations | | Turnover Intention | | Occupational Self-Efficacy | |  |  |
| --- | --- | --- | --- | --- | --- | --- | --- | --- | --- | --- | --- | --- | --- | --- | --- | --- |
| MIMI Total | | 1.000 | | .684^**^ | | .976^**^ | | .372^**^ | | -.290^**^ | | .188^**^ | | -.182^**^ | | |
| Microinsults | | .684^**^ | | 1.000 | | .560^**^ | | .302^**^ | | -.185^**^ | | .085 | | -.194^**^ | | |
| Microinvalidations | | .976^**^ | | .560^**^ | | 1.000 | | .346^**^ | | -.284^**^ | | .189^**^ | | -.166^**^ | | |
| *Note*: * p < .05. ** p < .01. | | | | | | | | | | | | | | | |  |
